# Characterization of dopamine D_2_ receptor coupling to G proteins in *postmortem* brain of subjects with schizophrenia

**DOI:** 10.1007/s43440-021-00305-4

**Published:** 2021-07-01

**Authors:** Iker Egusquiza, Eva Munarriz-Cuezva, Rafael Segarra, Javier González-Maeso, Luis F. Callado, J. Javier Meana, Rebeca Diez-Alarcia

**Affiliations:** 1 Department of Pharmacology, University of the Basque Country UPV/EHU, 48940 Leioa, Bizkaia Spain; 2 Centro de Investigación Biomédica en Red de Salud Mental, CIBERSAM, Leioa, Spain; 3 Biocruces Bizkaia Health Research Institute, Barakaldo, Bizkaia Spain; 4 Department of Neurosciences, University of the Basque Country UPV/EHU, Leioa, Bizkaia Spain; 5 Department of Physiology and Biophysics, School of Medicine, Virginia Commonwealth University, Richmond, VA USA

**Keywords:** D_2_ receptors, Schizophrenia, G protein, Human brain, [^35^S]GTPγS, Dopamine

## Abstract

**Background:**

Alterations of dopamine D_1_ (D1R) and D_2_ receptor (D2R) are proposed in schizophrenia but brain neuroimaging and *postmortem* studies have shown controversial results in relation to D1R and D2R density. Besides, scarce information on the functionality of brain D1R and D2R is available. The present study characterized G-protein activation by D1R and D2R agonists in *postmortem* human brain. Furthermore, D2R functional status was compared between schizophrenia and control subjects.

**Methods:**

G-protein receptor coupling was assessed in control caudate nucleus and frontal cortex by [^35^S]GTPγS-binding stimulation induced by increasing concentrations (10^–10^–10^–3^ M) of dopamine, and the selective dopaminergic agonists SKF38393 (D1R) and NPA (D2R). Concentration–response curves to NPA stimulation of [^35^S]GTPγS binding were analyzed in antipsychotic-free (*n* = 10) and antipsychotic-treated (*n* = 7) schizophrenia subjects and matched controls (*n* = 17).

**Results:**

In caudate, [^35^S]GTPγS-binding responses to agonists were compatible with the existence of functional D2R. In contrast, stimulations in cortex showed responses that did not correspond to D1R or D2R. [^35^S]GTPγS-binding activation by NPA in caudate displayed biphasic curves with similar profile in schizophrenia (EC_50H_ = 7.94 nM; EC_50L_ = 7.08 μM) and control (EC_50H_ = 7.24 nM; EC_50L_ = 15.14 μM) subjects. The presence or absence of antipsychotic medication did not influence the pharmacological parameters.

**Conclusions:**

Feasibility of functional evaluation of dopamine receptors in *postmortem* human brain by conventional [^35^S]GTPγS-binding assays appears to be restricted to signalling through inhibitory G_i/o_ proteins. These findings provide functional information about brain D2R status in subjects with schizophrenia and do not support the existence of D2R supersensitive in this mental disorder.

**Supplementary Information:**

The online version contains supplementary material available at 10.1007/s43440-021-00305-4.

## Introduction

Schizophrenia is a chronic mental syndrome, characterized by a wide range of symptoms that include hallucination, delusions, disorganized thinking, anhedonia, apathy and cognitive deficits, among others. Despite important efforts, the etiopathogenesis of this disorder remains elusive. The most extended hypothesis of schizophrenia proposes that hyperactivity of dopamine transmission in the mesolimbic neurotransmission system is responsible for positive symptoms (hallucinations, delusions), whereas a prefrontal hypodopaminergia would contribute to negative (alogia, anhedonia, abolition) and cognitive symptoms [1]. This hypothesis was conceptualized from the seminal work of Carlsson and Lindqvist who identified the alterations of dopamine turnover induced by antipsychotic drugs [2], and the additional evidence that amphetamine, which increases monoamine synaptic concentrations, exacerbated psychotic symptoms [3]. The subsequent demonstration that potency of antipsychotic drugs to block dopamine D_2_ receptors correlates with their effective clinical doses [4] provided credibility to the excess of dopaminergic activity as an underlying mechanism of psychosis in schizophrenia.

Dopamine receptors are members of the G-protein-coupled receptor (GPCR) superfamily and conventionally subdivided into D_1_ and D_2_ families. The D_1_ family (D_1_ and D_5_ receptors) is coupled to stimulatory G_s_ proteins, which activate adenylyl cyclase, while the D_2_ family (D_2_, D_3_ and D_4_ receptors) inhibits adenylyl cyclase activity by interacting with G_i/o_ proteins. Alternative effector pathways have also been described for dopamine receptor signalling [5]. In the central nervous system (CNS), dopamine D_2_ receptors (D2R) are expressed pre- and post-synaptically and mainly distributed in striatum, nucleus accumbens and olfactory tubercle, with minor expression in the substantia nigra, ventral tegmental area, hypothalamus, cortex, amygdala and hippocampus. Dopamine D_1_ receptors (D1R) are post-synaptic receptors and highly expressed in striatum, amygdala, olfactory bulb and frontal cortex [5].

In vivo neuroimaging based on positron emission tomography (PET) and single photon emission computerized tomography (SPECT) techniques has been used to evaluate the status of D2R and D1R in schizophrenia patients. Initial studies in basal ganglia were inconsistent, with some of them reporting increased D2R density and others no differences from controls [1, 6]. Elevated D2R density was suggested to be a consequence of receptor upregulation after long-term antipsychotic medication since drug-naïve patients did not show such PET alterations [7]. In vitro* postmortem* binding studies corroborated the finding of enhanced striatal D2R-binding density in schizophrenia [8, 9]. As for D2R, in vivo observations on D1R density in prefrontal cortex of drug-naïve schizophrenia patients have shown discrepancies [6]. In contrast to this earlier focus on postsynaptic D1R and D2R dysregulation, more recent findings point towards a critical role for presynaptic dopamine dysfunction in schizophrenia. Thus, elevated presynaptic dopamine synthesis capacity [10, 11], higher synaptic dopamine concentration [12, 13], and amphetamine-induced dopamine release [11, 14] have been demonstrated in striatum of subjects with schizophrenia. Conversely, evidence suggests a reduction of amphetamine-induced dopamine release in frontal cortex of schizophrenia patients [15], indicating a presynaptic hypodopaminergia in this brain area [15].

According to the ternary model, GPCRs display two interchangeable conformations in equilibrium. The high-affinity receptor state coupled to heterotrimeric G proteins possesses higher affinity for endogenous and exogenous agonists, and represents the functional active conformation. Otherwise, the low-affinity receptor state is uncoupled from G proteins and shows preferential affinity for inverse agonists. Antagonist drugs do not discriminate between the high- and low-affinity states, labelling with similar affinity both conformational states. Application of this pharmacological concept could shed light on the multiple and conflicting results about D2R in schizophrenia. In this context, an elevation of D2R in the high-affinity conformation in the caudate, putamen and nucleus accumbens of schizophrenia subjects has been shown [16]. However, signalling studies to assess the functional status of this receptor in this disorder are absent.

The first design of the present study sought to evaluate the functional status of dopamine-sensitive receptors in brain caudate nucleus and frontal cortex of subjects with schizophrenia and their matched controls. A basic pharmacological characterization of G-protein responses to dopamine receptor activation was initially performed to explore the feasibility of the study in both human brain areas. The functional coupling of dopamine receptors to G proteins was assessed by [^35^S]GTPγS-binding assays as previously described [17–19]. Assays of [^35^S]GTPγS-binding responses to the endogenous neurotransmitter dopamine and to selective D1R and D2R synthetic agonists were performed under similar methodological conditions. The method is based on the ability of the radiolabelled GTP non-hydrolyzable analog called sulfur 35 labelled guanosine-5’-*O*-(γ-thio)-triphosphate, [^35^S]GTPγS, to specifically bind Gα subunits of G proteins. Activation of GPCRs by agonists promotes the GDP/GTP exchange and, therefore, facilitates the binding of [^35^S]GTPγS to the different α subunits of G proteins. Thus, the agonist-induced modulation of the [^35^S]GTPγS binding provides a functional measure of GPCR functionality. This assay has been widely used as a functional approach in *postmortem* brain tissue studies, although it has the limitation that does not work equally well for all the G-protein subtypes [20, 21]. In spite of that, several studies in different psychiatric disorders have demonstrated the feasibility of the [^35^S]GTPγS binding technique to detect altered responses of different GPCRs in *postmortem* brain tissue of affected subjects [22–29].

## Materials and methods

Human brain samples were obtained at autopsy in the Basque Institute of Legal Medicine, Bilbao, Spain, and stored at -80ºC in the brain bank facilities of the University of the Basque Country until assay. A toxicological screening on blood at the time of death (detection of antidepressants, antipsychotics, psychotropic drugs and ethanol) was performed at the National Institute of Toxicology, Madrid, Spain. Collection and use of the samples were developed in compliance with Spanish policies of research in *postmortem* brain studies and were approved by the ethical review board of the University of the Basque Country.

Specimens of the dorsolateral prefrontal cortex and the caudate nucleus head were selected from 17 subjects with *antemortem* diagnosis of schizophrenia according to DSM-IV and DSM-IVTR criteria, and from 17 matched control subjects. Seven additional control subjects were selected to perform the initial characterization of [^35^S]GTPγS binding assays. The schizophrenia subjects were divided into antipsychotic-free and antipsychotic-treated according with the presence or absence of antipsychotics in blood at the time of death. Presence of ethanol and drugs of abuse in control subjects was not considered as exclusion criteria due to their regular presence among schizophrenia subjects. Schizophrenia and control subjects were matched for age, sex, and *postmortem* delay (time interval between death and autopsy). All these parameters were not statistically different between groups. Demographic characteristics, *postmortem* delay and toxicological information of subjects with schizophrenia and individually matched controls are shown in Table 1.

**Table 1:** Demographic characteristics, *postmortem* delay, and toxicological information of individual cases of schizophrenia subjects and their respective matched controls

	Gender (F/M)	Age (years)	*Postmortem* delay (h)	Blood toxicology
Case 1	M	21	24	(−)
Control 1	M	21	30	Amp, THC, OH
Case 2	M	30	51	(−)
Control 2	M	29	18	(−)
Case 3	M	44	7	Clot, Lmep, Nor, Bip
Control 3	M	44	23	(−)
Case 4	M	30	18	Ola
Control 4	M	30	11	THC
Case 5	M	29	6	THC
Control 5	M	29	36	OH
Case 6	M	31	14	Lor
Control 6	M	32	28	Amp, OH
Case 7	M	32	8	Quet, Lor
Control 7	M	32	20	OH
Case 8	M	48	20	(−)
Control 8	M	47	18	Dia
Case 9	M	23	16	Sul
Control 9	M	23	17	(−)
Case 10	M	35	3	Quet, Nor
Control 10	M	36	23	(−)
Case 11	F	30	28	Hal,
Control 11	F	29	31	(−)
Case12	M	31	11	(−)
Control 12	M	31	13	OH
Case 13	M	33	14	Dia
Control 13	M	33	4	(-)
Case14	M	45	3	Nor
Control 14	M	44	21	(−)
Case 15	F	37	58	Mor, Dia
Control 15	F	36	38	Mor, Dia, Coc
Case 16	M	46	22	Bip
Control 16	M	46	24	(−)
Case 17	M	35	11	Cloz, Nor
Control 17	M	36	18	Coc, OH

*F* female, *M* male. The *postmortem* delay represents the time elapsed between death and autopsy. Toxicological results are coded as amphetamine (Amp), biperiden (Bip), cannabis (THC), clotiapine (Clot), clozapine (Cloz), cocaine and benzoilecgonine (Coc), diazepam (Dia), ethanol (OH), haloperidol (Hal), levomepromazine (Lmep), lorazepam (Lor), morphine (Mor), nordiazepam (Nor), olanzapine (Ola), quetiapine (Quet), sulpiride (Sul)

The membrane preparation and the [^35^S]GTPγS binding assays were performed as previously reported in detail [17, 18]. P_2_ membrane fractions (40 μg) were incubated at 30 °C for 2 h (1 mM EGTA, 3 mM MgCl_2_, 100 mM NaCl, 1 mM DTT, 50 μM GDP, 50 mM Tris–HCl at pH 7.4) with 0.5 nM [^35^S]GTPγS. Increasing concentrations of the endogenous neurotransmitter dopamine (10^–10^–10^–3^ M), the selective D1R agonist SKF38393 (10^–10^–10^–3^ M) or the selective D2R agonist *N*-propylapomorphine (NPA) (10^–10^–10^–3^ M) were added to delineate receptor-stimulated [^35^S]GTPγS binding curves. The pharmacological profile of the targets was tested by using the selective D1R antagonist SCH23390 (10 μM) or the D2R antagonist haloperidol (10 μM). Basal binding was assumed to be the specific [^35^S]GTPγS binding in absence of agonist. Nonspecific [^35^S]GTPγS binding was determined in the presence of unlabelled GTPγS (10 μM) and represented around 2% of total binding. The reaction was terminated by rapid vacuum through Whatman GF/C glass fibre filters and the remaining bound radioactivity was measured by liquid scintillation counting. Specific [^35^S]GTPγS binding values were calculated subtracting nonspecific-binding values. Then, these specific values were transformed to percentage of basal binding ([^35^S]GTPγS binding values in the absence of any exogenous drug) which was considered as 0%. Results are represented as increases of percentage over the respective basal values.

Individual concentration–response curves of the agonist-stimulated [^35^S]GTPγS binding assays were analyzed by non-linear regression using GraphPad Prism software. Statistical selection between monophasic or biphasic curve models was made by the extrasum-of-square *F* test, using GraphPad Prism. The maximal stimulation expressed as percentage over the corresponding basal binding ([^35^S]GTPγS binding in the absence of any exogenous ligand) and the concentration of the agonist that elicited half-maximal effect (EC_50_) were obtained. EC_50_ values were normalized as log EC_50_ (-pEC_50_) values before parametric comparisons. Student’s *t* test was used to compare individual values of pharmacological parameters between schizophrenia and control groups. On the other hand, non-linear regression co-analysis of all individual curves of each group was conducted to further compare schizophrenia vs. control groups, and antipsychotic-free or antipsychotic-treated subjects vs. corresponding matched controls. The analysis that permitted one or more of the parameters to be shared without a significant increase in the residual variance was taken as the best fit [30]. Results are expressed as mean ± standard error (SE) and the level of statistical significance was chosen as *p* = 0.05.

Sulfur 35 labelled guanosine-5’-*O*-(γ-thio)-triphosphate ([^35^S]GTPγS; 1250 Ci/mmol) was purchased from PerkinElmer Life Science (Maanstraat, Germany). Dopamine HCl, GDP, GTPγS, haloperidol and R(−)-propylnorapomorphine HCl (also termed *N*-propylapomorphine) were obtained from Sigma Aldrich (St. Louis, USA). R( +)-SCH23390 HBr, ( ±)-SKF38393 HBr and MDL11939 were purchased from Tocris Bioscience (Bristol, UK). All other chemical reagents were of the highest analytical quality and were obtained from Merck (Darmstadt, Germany) or Sigma-Aldrich.

## Results

**Fig. 1:**
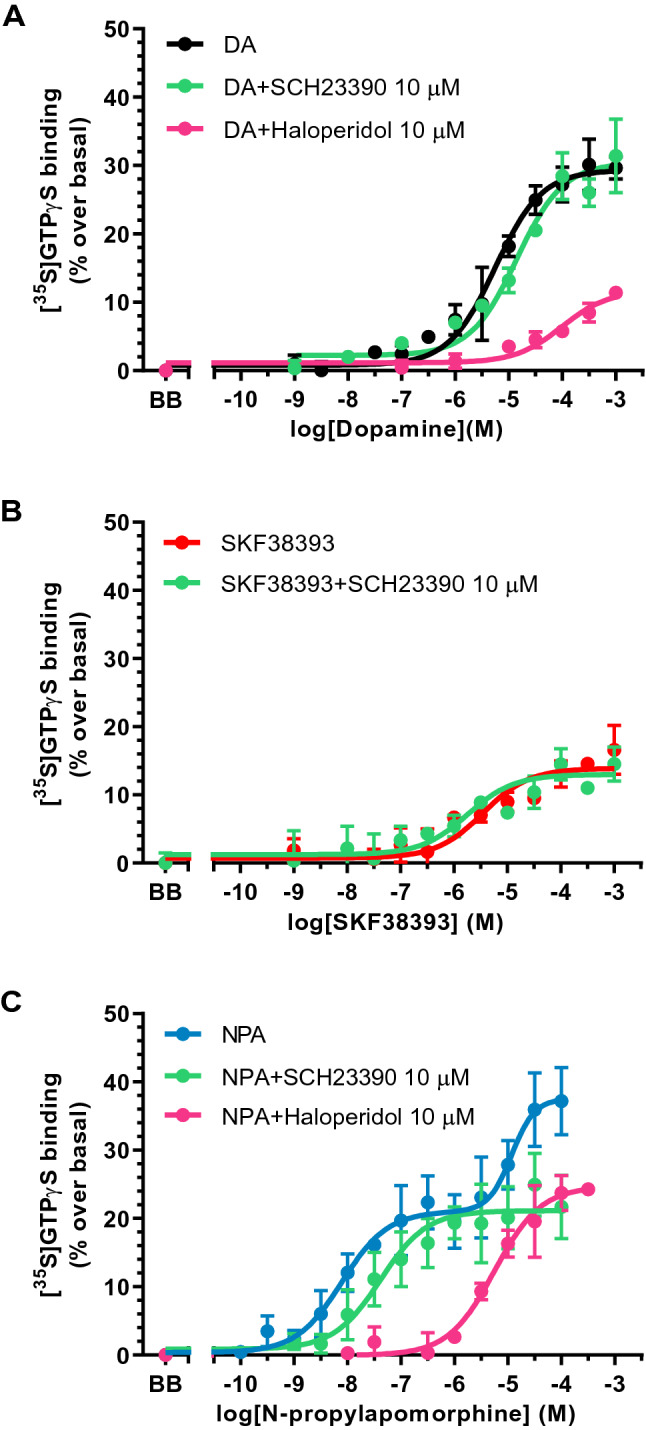
Concentration response curves of the [^35^S]GTPγS binding stimulation by increasing concentrations (10^–10^-10^–3^ M, 13 concentrations) of **A** dopamine (DA), **B** the selective D1R agonist SKF38393 and **C** the selective D2R agonist *N-*­propylapomorphine (NPA) in absence and presence of the D1R antagonist SCH23390 (10 μM) or the D2R antagonist haloperidol (10 μM). Experiments were performed in human caudate of 4–7 different control subjects. Points are mean ± standard error of the mean and represent the increase (in percentage) over respective basal values. BB represents the basal binding in absence of agonist

The concentration–response curves of [^35^S]GTPγS binding for dopamine in caudate head membrane preparations displayed monophasic stimulation curves with an estimated EC_50_ = 5.57 μM (Table 2). The selective D1R antagonist SCH23390 (10 μM) did not modify the stimulation parameters (*t*_10_ = 2.16, *p* = 0.06), whereas the D2R antagonist haloperidol (10 μM) promoted a right-shift of the curves (*t*_9_ = 6.43, *p* = 0.0001) that did not reach a clear asymptote (Table 2). These findings were compatible with a main dopamine-induced stimulation of G proteins that could be mediated by D2R (Fig. 1A).

**Table 2:** Pharmacological parameters of the concentration–response curves of [^35^S]GTPγS binding stimulation induced by different agonists in absence and presence of selective antagonists in human caudate

	log EC_50H_	log EC_50L_	*E*_max_ (%)	Fraction H (%)
Dopamine	− 5.25 ± 0.10	–	29 ± 1	N/A
Dopamine + haloperidol	− 4.01 ± 0.19	–	12 ± 1^a^	N/A
Dopamine + SCH23390	− 4.85 ± 0.17	–	30 ± 2	N/A
SKF38393	− 5.46 ± 0.23	–	14 ± 1	N/A
SKF38393 + SCH23390	− 5.63 ± 0.33	–	12 ± 1	N/A
*N*-propylapomorphine (NPA)	− 8.10 ± 0.28	− 4.93 ± 0.23	38 ± 4	56 ± 11
NPA + haloperidol	–	− 5.25 ± 0.14	25 ± 2	N/A
NPA + SCH23390	− 7.39 ± 0.26	–	21 ± 1	N/A
NPA + MDL11939	− 8.03 ± 0.21	− 4.76 ± 0.30	37 ± 3	54 ± 8

Values are means ± SE of independent experiments performed in tissue homogenates from 4–7 different control subjects. logEC_50_ values are log normalized values of the agonist concentration that elicited half-maximal effect obtained from concentration–response curves. E_max_ is the maximal % stimulation of [^35^S]GTPγS binding over basal values. H and L represents the high-, and low-affinity fractions of the curves, respectively. N/A: not applicable due to the existence of a monophasic curve model. ^a^E_max_ for dopamine + haloperidol represents an approximate estimation due to absence of asymptotic values

The concentration response curves to the selective D1R agonist SKF38393 in caudate were also monophasic with low stimulatory effect and estimated EC_50_ = 3.46 μM (Table 2). The selective D1R antagonist SCH23390 (10 μM) was not able to displace the stimulations induced by SKF38393 in this area (*t*_6_ = 0.42, *p* = 0.69), which suggests that this effect was not mediated by D1R (Fig. 1B).

When the selective D2R agonist NPA was used to stimulate the [^35^S]GTPγS binding, biphasic concentration–response curves [*F*(2,103)  = 20.17, *p* < 0.0001 vs. monophasic model] were obtained with EC_50_ values of 6.31 nM and 85.11 μM (Table 2). The NPA stimulations were also carried out in the presence of SCH23390 (10 μM) and, then, monophasic curves corresponding to the high-potency component of NPA stimulation were obtained with a non-significant slight displacement of the curve to the right (EC_50_ = 41 nM) (*t*_10_ = 1.78, *p* = 0.11) (Table 2). Monophasic curves were also obtained in the presence of haloperidol (10 μM), but this time potency values mirrored the low-potency component of NPA stimulation (EC_50_ = 5.59 μM) (*t*_10_ = 1.07, *p* = 0.31), whereas the high-potency component was abolished (*t*_10_ = 6.84, *p* < 0.0001) (Table 2). These results indicated that the high-potency component of the [^35^S]GTPγS binding stimulation by NPA was compatible with D2R (Fig. 1C). The low-potency component of NPA stimulation could apparently be sensitive to SCH23390, suggesting the presence of D1R-mediated activity but other possibilities should also be considered due to the elevated concentration of SCH23390 used.

Since SCH23390 displays important affinity (9.3–30 nM) [31, 32] and efficacy [33] on serotonin 5-HT_2_ receptors, the hypothesis that the low-potency component of NPA stimulation in caudate represents a serotonin 5-HT_2A_ receptor interaction was tested. The selective 5-HT_2A_ receptor antagonist MDL11939 (1 μM) did not modify the biphasic stimulatory curves promoted by NPA on [^35^S]GTPγS binding (*t*_8_ = 0.15, *p* = 0.88 for the NPA high potency; *t*_8_ = 0.42, *p* = 0.69 for the NPA low potency), which dismissed the idea (Table 2 and Supplementary Fig. 1).

**Fig. 2:**
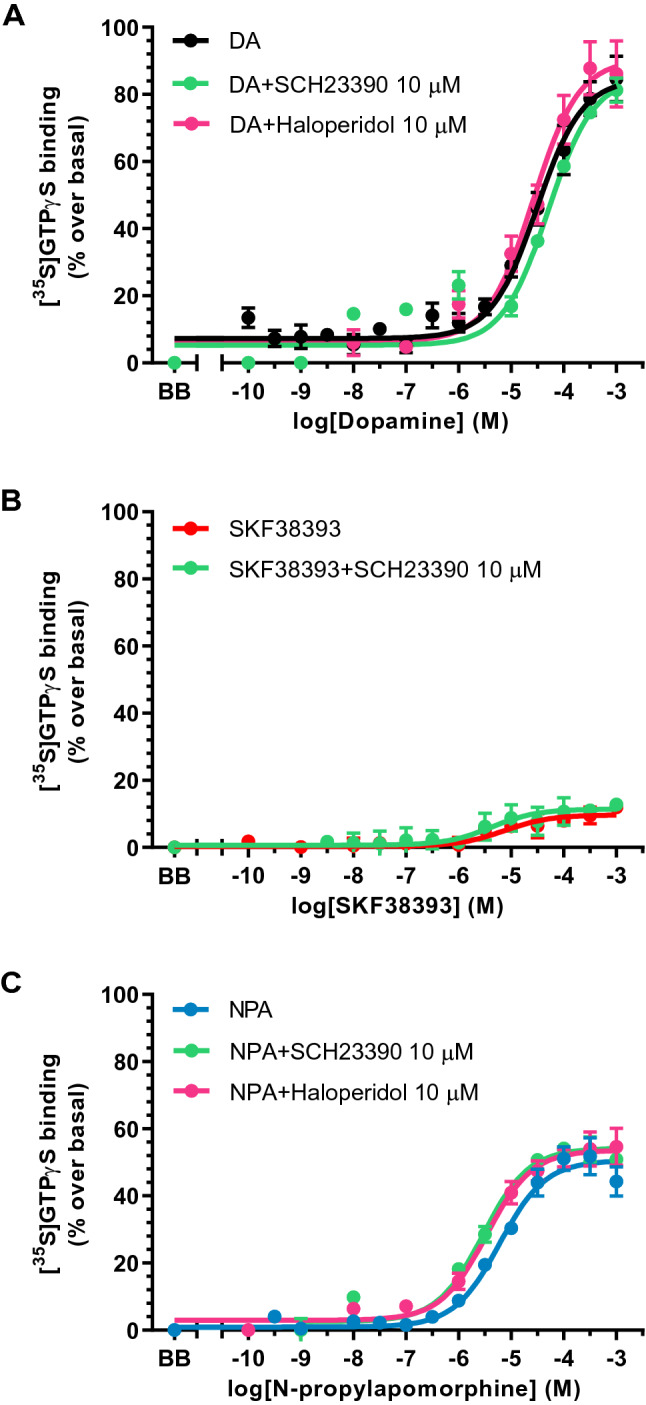
Concentration response curves of the [^35^S]GTPγS binding stimulation by increasing concentrations (10^–10^-10^–3^ M, 13 concentrations) of **A** dopamine (DA), **B** the selective D1R agonist SKF38393 and **C** the selective D2R agonist *N-*­propylapomorphine (NPA) in absence and presence of the D1R antagonist SCH23390 (10 μM) or the D2R antagonist haloperidol (10 μM). Experiments were performed in human frontal cortex of 3–7 different control subjects. Points are mean ± standard error of the mean and represent the increase (in percentage) over respective basal values. BB represents the basal binding in absence of agonist

In *postmortem* human frontal cortex membrane preparations, the concentration–response curves of dopamine on [^35^S]GTPγS binding showed a monophasic pattern with an estimated EC_50_ = 30.4 μM (Table 3). Neither SCH23390 (10 μM) (*t*_5_ = 1.52, *p* = 0.19) nor haloperidol (10 μM) (*t*_8_ = 0.24, *p* = 0.82) modified the dopamine-induced stimulation curves (Table 3). Compared with values in caudate, EC_50_ values of dopamine curves were lower (*t*_10_ = 1.78, *p* = 0.11) and maximal effects were higher (*t*_10_ = 11.5, *p* < 0.0001) in frontal cortex. These findings were compatible with a dopamine activity on G proteins that was not mediated by either D1R or D2R (Fig. 2A).

**Table 3:** Pharmacological parameters of the concentration–response curves of [^35^S]GTPγS binding stimulation induced by different agonists in absence and presence of selective antagonists in human frontal cortex

	log EC_50_	*E*_max_ (%)
Dopamine	− 4.52 ± 0.10	85 ± 4
Dopamine + haloperidol	− 4.56 ± 0.12	90 ± 5
Dopamine + SCH23390	− 4.30 ± 0.10	85 ± 4
SKF38393	− 5.13 ± 0.27	10 ± 1
SKF38393 + SCH23390	− 5.38 ± 0.39	11 ± 2
*N*-propylapomorphine (NPA)	− 5.22 ± 0.10	51 ± 2
NPA + haloperidol	− 5.47 ± 0.11	54 ± 2
NPA + SCH23390	− 5.54 ± 0.10	54 ± 3

Values are means ± SE of independent experiments performed in tissue homogenates from 3–7 different control subjects. logEC_50_ values are log normalized values of the agonist concentration that elicited half-maximal effect obtained from concentration–response curves. *E*_max_ is the maximal % stimulation of [^35^S]GTPγS binding over basal values

SKF38393 stimulations of [^35^S]GTPγS binding in frontal cortex were very low with an estimated EC_50_ = 7.41 μM (Table 3). SCH23390 (10 μM) did not modified SKF38393 curves in this brain area (*t*_5_ = 0.55, *p* = 0.61). This fact contributed to reject the possibility of D1R involvement in the effect of SKF38393 (Fig. 2B).

In frontal cortex, NPA stimulation curves of the [^35^S]GTPγS binding were monophasic with EC_50_ value of 5.99 μM (Table 3). The D1R antagonist SCH23390 (10 μM) did not modify NPA curves (*t*_7_ = 2.23, *p* = 0.06) (Table 3). In the same way, NPA-mediated stimulation was insensitive to haloperidol (10 μM) (*t*_8_ = 1.64, *p* = 0.14) (Table 3). The EC_50_ values of NPA in the frontal cortex were in the micromolar range, which corresponds to values of the low-potency component of NPA curves in caudate (Fig. 2C). These results suggested that the [^35^S]GTPγS-binding stimulation by NPA in frontal cortex did not represent activity on D1R or D2R, and could involve alternative GPCRs (Fig. 2C), as shown with dopamine (see Fig. 2A).

**Fig. 3:**
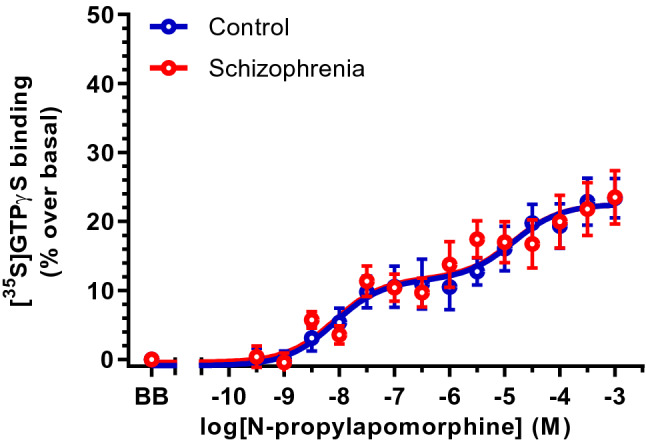
Concentration response curves of the [^35^S]GTPγS binding stimulation by increasing concentrations (10^–10^-10^–3^ M, 13 concentrations) of the selective D2R agonist *N*-propylapomorphine (NPA) in caudate of subjects with schizophrenia (*n* = 17) and matched controls (*n* = 17). Points are mean ± standard error of the mean and represent the increase (in percentage) over respective basal values. BB represents the basal binding in absence of agonist

After this pharmacological characterization of dopamine receptor targets involved in the functional responses induced by dopamine, SKF38393 and NPA, and to assess the status of caudate D2R in schizophrenia, concentration–response curves for NPA were performed in membrane homogenates from 17 subjects with schizophrenia and 17 individually matched controls (Table 1). Basal activity of [^35^S]GTPγS binding was not different between schizophrenia (1,952 ± 121 fmol/mg protein) and control (1,755 ± 80 fmol/mg protein) groups (*t*_32_ = 1.34, *p* = 0.19). NPA stimulatory curves better adjusted to a biphasic concentration-effect model in both groups. The co-analysis of the curves demonstrated their similarity [*F*(4,366) = 0.178; *p* = 0.95]. EC_50_ values (*t*_32_ = 0.06, *p* = 0.96 for the NPA high-potency; *t*_32_ = 0.55, *p* = 0.59 for the NPA low-potency), maximal stimulatory effects (*t*_32_ = 0.35, *p* = 0.73), and proportion of high/low components (*t*_32_ = 0.55, *p* = 0.59) did not differ between schizophrenia and control curves (Table 4; Fig. 3).

**Table 4:** Pharmacological parameters of the concentration–response curves of [^35^S]GTPγS binding stimulation induced by *N*-propylapomorphine (NPA) in human caudate of subjects with schizophrenia and controls

	log EC_50H_	log EC_50L_	*E*_max_ (%)	Fraction H (%)
Schizophrenia (*n* = 17)	− 8.11 ± 0.40	− 5.17 ± 0.49	22 ± 2	56 ± 10
Control (*n* = 17)	− 8.14 ± 0.37	− 4.82 ± 0.36	23 ± 2	49 ± 8

Values are means ± SE of independent experiments performed in tissue homogenates from 17 subjects in each group. logEC_50_ values are log normalized values of the agonist concentration that elicited half-maximal effect obtained from concentration–response curves. * E*_max_ is the maximal % stimulation of [^35^S]GTPγS binding over basal values. H and L represents the high-, and low-affinity fractions of the curves, respectively

The one-to-one matching design of the study allowed us to separately compare antipsychotic-free (*n* = 10) and antipsychotic-treated (*n* = 7) schizophrenia subjects with their respective controls. The NPA stimulation curve of [^35^S]GTPγS binding in antipsychotic-free subjects was not different to that in controls [*F*(4,276) = 2.02, *p* = 0.09]. Estimated EC_50_ values in antipsychotic-free schizophrenia group (EC_50H_ = 8.32 nM, EC_50L_ = 13.2 μM) were in similar range to those in matched controls (EC_50H_ = 4.79 nM, EC_50L_ = 24.6 μM). In a similar way, antipsychotic-treated schizophrenia subjects showed NPA-mediated [^35^S]GTPγS-binding stimulations that did not differ from that of matched controls (F[4,172] = 0.68, *p* = 0.61). EC_50_ values in antipsychotic-treated subjects (EC_50H_ = 3.80 nM, EC_50L_ = 1.48 μM) were similar to EC_50_ values in corresponding controls (EC_50H_ = 5.75 nM, EC_50L_ = 2.13 μM).

## Discussion

The present study focused on G-protein activation by D1R and D2R agonists in membrane homogenates obtained from *postmortem* human caudate and prefrontal cortex. As functional data for dopamine receptors in human brain are scarce in the literature, basic characterization of [^35^S]GTPγS binding responses to D1R and D2R activation by dopamine and subtype selective agonists was initially performed to explore the feasibility of the study in both human brain areas. Afterwards, the potential dysfunction of D2R coupling to G proteins in caudate of schizophrenia subjects was assessed. Caudate head was selected due to the important activation by D2R agonist [34] and the D2R density alterations in basal ganglia previously described in schizophrenia [1, 6, 7]. The dorsolateral prefrontal cortex was explored, because it constitutes a critical area in schizophrenia where D1R show higher expression than D2R [5].

Previous [^35^S]GTPγS-binding assays in rodent striatal membranes generally described the presence of D2R [35–37] in this area. Thus, stimulatory responses were obtained for quinpirole (pEC_50_ = 5.4–5.8), pergolide (pEC_50_ = 6.5–7.7) and NPA (pEC_50_ = 7.8–8.4), among other selective D2R agonists [35–37]. In contrast, the preferential D1R agonists SKF38393 (pEC_50_ = 3.2) and SKF81297 were almost inactive and, when small efficacy was displayed, insensitivity to the selective D1R antagonist SCH23390 was observed [37]. In rat striatum, concentration–response [^35^S]GTPγS-binding curves to the endogenous agonist dopamine revealed EC_50_ values in the micromolar range (pEC_50_ = 5.2–5.5). In these studies, the dopamine-induced stimulations were inhibited by the D2R antagonists haloperidol (pK_b_ = 9.4–8.1) and raclopride ((pK_b_ = 9.0–8.2) but not by the D1R antagonist SCH23390 both in striatal membranes [35–37] and in tissue sections [38].

In *postmortem* human brain tissue, previous autoradiographic mapping of [^35^S]GTPγS binding response to single concentrations of dopamine or selective D1R and D2R agonists demonstrated an anatomical distribution of the functional activation with the highest effects (24% approximately) in dorsal caudate [34]. Present findings in the same brain area indicated a D2R pharmacological profile that agrees with previous data in rodents. Dopamine stimulates [^35^S]GTPγS binding with EC_50_ values in the micromolar range and the curves are considerably shifted to the right by the D2R antagonist haloperidol but not by the D1R antagonist SCH23390. NPA evoked a biphasic stimulation of [^35^S]GTPγS binding in human caudate, and a similar profile compatible with D2R was observed for the high-potency component of the curves. Furthermore, the D1R agonist SKF38393 induced a lower [^35^S]GTPγS-binding stimulation that was insensitive to the D1R antagonist SCH23390. Therefore, the present results suggest a selective targeting of D2R-mediated activation of G proteins in human caudate. Several limitations must be acknowledged, including the absence of a full characterization of dopamine, SKF38393 and NPA targets. Both haloperidol and SCH23390 were used at high and non-selective D2R and D1R affinity concentrations, respectively. This may have led to antagonism over other GPCRs, especially when dopamine was used as agonist. It should also be recognized that maximal stimulatory effects observed herein are lower than those reported in rodents [35, 37], although similar to values in human caudate sections [34]. Efficacy of agonist-stimulated [^35^S]GTPγS binding is very sensitive to experimental conditions and mainly depends on GDP and NaCl concentrations employed [17, 19, 35–37]. Therefore, discrepancies in this parameter between studies may reflect particular incubation conditions of the different assays and/or differences between human and rodents.

In human frontal cortex, dopamine and NPA stimulation of [^35^S]GTPγS binding displayed monophasic curves with lower EC_50_ values and higher maximal stimulations than in caudate. The presence of haloperidol or SCH23390 did not attenuated the agonist-induced responses. Moreover, SKF38393 promoted a small stimulation with similar profile to caudate and that, again, was insensitive to SCH23390. Hence, the findings substantially reflect the role in the response of an undetermined GPCR different from D1R and D2R. These data are consistent with earlier findings in the same human brain area where dopamine elicited a low-potency response (pEC_50_ = 4.24) insensitive to both D1R and D2R antagonists but sensitive to the α_2_-adrenoceptor antagonist RX821002 (2-methoxyidazoxan) [19].

Besides coupling to G proteins that promote adenylyl cyclase activation or inhibition, it has been suggested the existence of a D1-like receptor that activates phospholipase C (PLC) and thus stimulates the production of diacylglycerol and inositol trisphosphate. The effector system for this non-canonical response is the G_q/11_-protein subtype [39]. The D1-like-mediated activation of G_q/11_ proteins has been found in rat cortex and striatum, among other brain areas [33, 40, 41]. The pharmacological evidence for the existence of this D1-like receptor is supported by the dopamine activity on this pathway, the functional agonist bias role of the D1R compound SKF83959 towards PLC signalling [42], and the antagonism elicited by SCH23390. Furthermore, it has been argued that this response would occur via coactivation of a D1R-D2R heterodimer functionally active in brain [43]. However, the existence of the D1R–D2R heterodimer is controversial [44] and the pharmacological activity of SKF83959 and related benzazepines as SKF38393 and SCH23390 seems to represent off-target effects due to their added affinity for serotonin 5-HT_2A/2C_ receptors and/or α_2_-adrenoceptors [31–33, 45]. According to this evidence, it is conceivable that [^35^S]GTPγS-binding stimulation by dopamine, NPA and SKF38393 observed in human frontal cortex in the present study represents activation of GPCRs unrelated to D1R and D2R. The exact pharmacological identity of this stimulation remains to be elucidated but is outside the scope of the present study.

In relation to the low-potency component of NPA stimulation in caudate, the present results discard a D1R-mediated component. The dopamine- and SKF38393-mediated stimulations of [^35^S]GTPγS binding were insensitive to SCH23390, which make this possibility unlikely. On the other hand, although there is no evidence in the literature of NPA affinity for 5-HT_2A_ receptors, the failure of NPA to detect this second fraction in the presence of SCH23390 and the above-mentioned affinity of this compound for 5-HT_2A_ receptors [32], led us to test whether the low-potency component of NPA represented activation of 5-HT_2A_ receptors. For this purpose, the selective 5-­HT_2A_ receptor antagonist MDL11939 was added to the NPA-mediated stimulation assay. MDL11939 did not modify the [^35^S]GTPγS-binding response to NPA in caudate, confirming the lack of 5-HT_2A_ receptor involvement in the biphasic stimulation.

[^35^S]GTPγS binding technique represents a functional approach that has been successfully applied to the detection of receptor-dependent G-protein activation by different GPCRs even in *postmortem* human brain tissue [17, 19, 46]. Guanine nucleotide exchange represents the first measurable step in the receptor-initiated signal transduction pathways, helping to avoid cross-talk interactions that affect the response. One limitation of both the conventional [^35^S]GTPγS binding assay using filtration techniques and functional autoradiography assays is the fact that appear to be restricted for detection of activation responses involving inhibitory G protein subtypes [21, 47–49]. Functional activation of other G proteins (e.g., G_s/olf_ and G_q/11_) in brain seems to be undetectable with this approaches [21]. The higher feasibility of detection of [^35^S]GTPγS binding to G_i/o_ proteins is attributed to two main reasons: (a) the relative abundance of G_i_ and specially G_o_ proteins in brain over other families [50], and (b) their higher rates of nucleotide exchange [47, 48]. Regarding dopamine receptors, D1R family is preferentially coupled to stimulatory G_s_ proteins, whereas D2R family mediates activation of G_i/o_ proteins [5]. In this context, and although D1R predominate over D2R family in frontal cortex, the pharmacological profile of G-­protein activation obtained in the present study for dopamine and D1R selective agonist in this area does not correspond to D1R and would be considered more probably as an off-target pharmacological effect. This fact also indicates that the methodological conditions for conventional [^35^S]GTPγS-binding activation herein used, are not the most appropriate to detect G-protein activation by D1Rs.

In summary, the findings of the present work confirm that feasibility to quantify the dopamine receptor-mediated activation of G proteins in brain is limited to D2R family. The study provides suitable methodological conditions to assess the functional status of D2R in caudate from subjects with schizophrenia. Alternative methods based on [^35^S]GTPγS-binding stimulations combined with immunoprecipitation assays by selective antibodies for the different G proteins could help to improve detection of D1R family functional activity in human brain [51].

The dopamine hypothesis of schizophrenia is based on the idea of an increased dopamine neurotransmission in brain. This enhanced activity may be mediated by increased neurotransmitter synthesis [10, 11], release [11, 14], synaptic concentrations [12, 13] and/or the existence of supersensitive postsynaptic dopamine receptors, especially D2R, the common target of antipsychotics. In schizophrenia patients, in vivo neuroimaging studies by PET and SPECT have reported controversial results in relation to D2R density. Early observations provided evidence for elevated binding capacity that argued in favour of the striatal hyperdopaminergic hypothesis. In contrast, further studies in drug naïve patients showed equivalent density to controls [1, 6, 16]. Discrepancies seem to be due to the low selectivity of first radiotracers ([^11^C]NSMP) compared to the most recents ([^11^C]raclopride and [^123^I]IBZM), and to the confounding effect of antipsychotic treatment [6]. *Postmortem* radioligand-binding studies have shown enhanced D2R density in caudate nucleus [8, 52], and the existence of heterogeneous subject populations, despite the shared diagnosis of schizophrenia [8, 9]. A large body of evidence describes supersensitivity of D2R as a consequence of effective antipsychotic treatment [53, 54]. As drug-free populations are difficult to obtain for *postmortem* studies, most studies do not include them and, in consequence, increased density of striatal D2R is the most reported finding in the literature.

Nevertheless, Seeman updated the dopaminergic hypothesis and proposed that schizophrenia is associated with striatal D2R supersensitivity due to disequilibrium between the high- (D2High) and the low- (D2Low) affinity conformations of the receptor [7, 16]. The term D2High represents the functional state of the receptor, which shows relative selectivity for agonist drugs and is coupled to G proteins. According to the hypothesis, an imbalance of D2R towards D2High might be present in schizophrenia, leading to G-protein over activation. In this context, the use of agonist radioligands for in vivo and in vitro studies of D2R in schizophrenia would offer advantage over antagonists, which do not discriminate between D2High and D2Low conformations. Consistent with this interpretation, antibody immunodetection of D2R, that do not discriminate among receptor conformations, in caudate of schizophrenia subjects with and without residual antipsychotics showed similar protein expression to controls [55]. Equivalent results were found in human frontal cortex [56]. In this context, the [^35^S]GTPγS-binding stimulation by selective D2R agonists would be useful for detecting specific alterations of the functional receptor conformation (D2High) associated to schizophrenia. The current study describes that assessment of striatal [^35^S]GTPγS-binding response to NPA displays overlapped curves in schizophrenia and control groups. This finding suggests the absence of striatal D2R hyperactivity in subjects with schizophrenia, at least in their coupling to G_i/o_ proteins. However, dysfunctions in alternative G-protein-independent D2R signalling pathways cannot be discarded [57]. In contrast to this result in brain, olfactory neuroepithelial cells from patients with schizophrenia displayed enhanced [^35^S]GTPγS binding to G_s/olf_ and G_i/o_ proteins in response to a single concentration of dopamine [58]. In absence of pharmacological characterization, this change in olfactory cells does not directly reflect selective D2R dysfunction and might be influenced by the antipsychotic treatment present in six out of the ten patients evaluated.

The presence or absence of antipsychotic treatment is a condition that influences D2R analysis. As previously mentioned, long-term antipsychotic administration upregulates D2R density and function [52–54]. In the present study, and to unmask a possible effect of antipsychotic treatment, independent and well-matched groups of antipsychotic-free and antipsychotic-treated subjects were defined and independently analyzed *vs*. respective control groups. Absence of changes in NPA activation of [^35^S]GTPγS binding was observed regardless presence or absence of antipsychotic treatment at death.

In summary, the present study provides functional information related to D2R status in *postmortem* brain of subjects with schizophrenia. The findings do not support the existence of supersensitive D2R alterations underlying hyperactive dopaminergic neurotransmission as a key feature of this mental disorder. Alternative formulations for the dopaminergic hypothesis of schizophrenia warrants further exploration.

## Supplementary Information

Below is the link to the electronic supplementary material.

Supplementary file1 (DOCX 38 KB)

## Abbreviations

CNS: Central nervous system
D1R: Dopamine D_1_ receptor
D2R: Dopamine D_2_ receptor
D2High: High-affinity state of dopamine D_2_ receptors
D2Low: Low-affinity state of dopamine D_2_ receptors
GPCR: G-protein coupled receptor
[^35^S]GTPγS: Sulfur 35 labelled guanosine-5′-*O*-(γ-thio)-triphosphate
NPA: R(−)-propylnorapomorphine, *N*-propylapomorphine
PET: Positron emission tomography
PLC: Phospholipase C
SPECT: Single photon emission computerized tomography
