# Supplementary material for: Characterization of dopamine D2 receptor coupling to G proteins in postmortem brain of subjects with schizophrenia
Source: Pharmacol Rep. 2021 Jul 1;73(4):1136–46. doi: 10.1007/s43440-021-00305-4 (PMC8413194; doi:10.1007/s43440-021-00305-4)
Supplement: Supplementary file 1 — Supplementary file1 (DOCX 38 KB) [file 43440_2021_305_MOESM1_ESM.docx]

**Characterization of dopamine D_2_ receptor coupling to G-proteins in *postmortem* brain of subjects with schizophrenia**

Iker Egusquiza^1^**^*^**, Eva Munarriz-Cuezva^1,2^**^*^**, Rafael Segarra^2,3,4^, Javier González-Maeso^5^, Luis F. Callado^1,2,3^, J. Javier Meana^1,2,3^, Rebeca Diez-Alarcia^1,2,3^

**Supplementary Figure 1**

Concentration response curves of the [^35^S]GTPγS binding stimulation by increasing concentrations (10^-10^-10^-3^ M, 13 concentrations) of the selective D2R agonist N-propylapomorphine (NPA) in absence of presence of the serotonin 5-HT_2A_ receptor antagonist MDL11939 (1 μM). Experiments were performed in human caudate of four different control subjects. Points are mean±standard error of the mean and represent the increase (in percentage) over respective basal values. BB represents the basal binding in absence of agonist.
